# Locally available agroresidues as potential sorbents: modelling, column studies and scale-up

**DOI:** 10.1186/s40643-021-00387-1

**Published:** 2021-04-28

**Authors:** Arth Jayesh Shah, Bhavin Soni, Sanjib Kumar Karmee

**Affiliations:** Thermo-Chemical Conversion Technology Division, Sardar Patel Renewable Energy Research Institute (SPRERI), Vallabh Vidyanagar, 388 120 Anand, Gujarat India

**Keywords:** Biosorbents, Circular economy, Waste management, Modelling, Scale up, Column study

## Abstract

Sawdust, cotton stalk and groundnut shell were used for removal of methylene blue from aqueous solution using batch sorption. Effect of initial dye concentration, temperature, and particle size of sorbents on methylene blue removal was investigated. Sorption capacity increases with rise in initial dye concentration and temperature. Impact of particle size on sorption of methylene blue was investigated and indicated that removal of dye increases with decrease in particle size of sorbents. Maximum sorption for sawdust, cotton stalks and groundnut shell were 9.22 mg g^−1^, 8.37 mg g^−1^ and 8.20 mg g^−1^ respectively; at 60 °C and 100 ppm initial dye concentration. Sorption isotherms were analyzed using fundamental Freundlich isotherm. Subsequently, sips isotherm model was employed for better fitting. Kinetic study shows that, biosorption process is pseudo-second-order in nature. During the course of this study, adsorption dynamics revealed that film diffusion was key step for biosorption. In addition, thermodynamics of sorption was studied; and it was found that Gibbs free energy (∆*G*°) decreases with increase in temperature. Sawdust was found to be best among all the sorbents. Therefore, column studies and breakthrough curve modelling were performed using sawdust. Furthermore, it was estimated that a scaled-up column using sawdust can treat 6672 L of wastewater in 24 h with 80% efficiency.

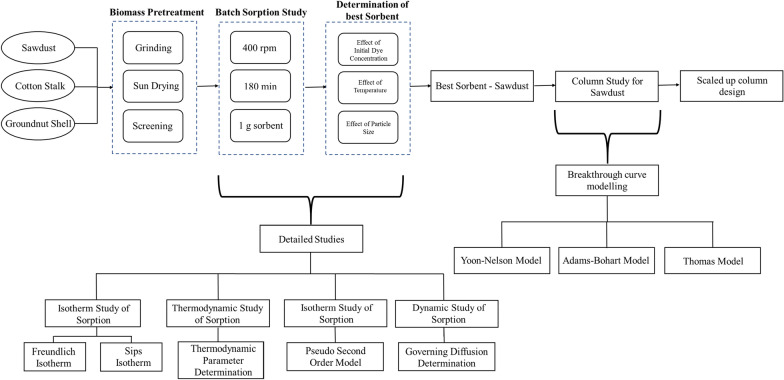

## Introduction

In general, stringent norms are laid down by monitoring and control authorities for chemical process industries (CPIs) for releasing effluents into the environment. To strictly follow the environmental policies, CPIs are developing and adopting different novel pollution controlling technologies for effluent release to control the deteriorating quality of water bodies. Removal of pollutant-dyes has always been a major concern for researchers involved in waste management; since their effective removal using conventional treatment processes is challenging. In this regard, modifications were performed in sewage treatment plants and wastewater treatment plants of CPIs to curb the dye contamination in the liquid effluent released by industries into the water bodies. Different dyes are released by industries processing papers, pulp, and paints. Many of these are carcinogenic in nature and toxic to humans and aquatic ecosystem (Acemioǧlu 2004). In addition, CPI’s man-made disasters like oil spills are known to cause devastating impacts on holistic biodiversity. Therefore, adequate measures needs to be taken to control these disastrous environmental consequences.

In the above context, activated carbon sorption, ion exchange, chemical coagulation and electrolysis are some of the potential techniques often adopted for dye removal from effluents (Nigam et al. 2000; An et al. 2001). Amongst these, activated carbon sorption is one of the most effective and commercially viable technology used extensively in waste water treatment plants of CPIs to remove dyes (McKay 1996; Mohan and Karthikeyan 1997; Chen et al. 2018). Nevertheless, activated carbon is expensive; therefore, it may be difficult for developing and underdeveloped countries to implement technologies based on activated carbons. Hence, there is an urgent need to develop technologies/processes for waste removal using low cost and readily available sorbents. Along this line, search for effective low-cost sorbents which can have similar effect as that of activated carbon became area of research for many researchers. In this regard, cotton stalks, cotton waste, cotton dust (Ertaş et al. 2010), sawdust (Garg et al. 2004; Azlina et al. 2013), orange peel (Namasivayam et al. 1996), banana peel (Annadurai et al. 2002), wood (Asfour et al. 1985; Ho and McKay 1998), bagasse pith (Nassar and El‐Geundi 1991), chitosan (Sakkayawong et al. 2005), and algae biochar (Chen et al. 2020a; Tan et al. 2020) are a few low-cost sorbents previously reported for dye removal and pollution control.

India is an agriculturally rich nation. Among various provinces of India; in particular, Gujarat is an agrarian state whose main crops include cotton, groundnut shell, rice, sugar cane, castor, tobacco and corn (Soni and Karmee 2020). These crops generate substantial amounts of residues. Around 5.5 million tonnes of surplus sawdust is generated annually in India and due to its easy availability, it can be employed as a sorbent (Soni and Karmee 2020). Similarly, ~ 8 lakh tonnes of groundnut shell and ~ 41 lakh tonnes of cotton stalks are produced annually in Gujarat (Kumari et al. 2020). These are low-cost, locally produced and readily available resources. Utilization of these locally available biomass residues as sorbents is important from circular economy, waste management, and sustainability point of views. Therefore, in this research sawdust, groundnut shell and cotton stalks were examined as low-cost bio-sorbents.

As mentioned earlier, several studies have been carried out in this area; however, a holistic approach that engrosses waste management and sustainable development strategies has not been applied widely yet at an industrial scale using locally available bioresidues. Hence, here, an effort towards implementation of this technology has been made by analyzing the sorbents behavior. In addition, emphasize is given on a scaled-up column design, which has the potential to cater future industrial needs.

## Materials and methods

### Materials

**Fig. 1:**
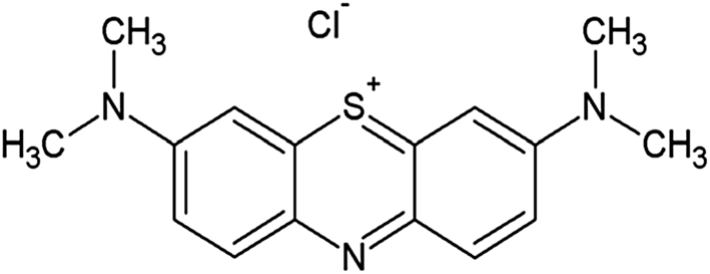
Chemical structure of methylene blue dye

A cationic dye methylene blue (MB) (microscopy grade) was procured from LOBA Chemie. The chemical structure of MB (a basic dye) containing secondary amine is presented in Fig. 1. Sawdust (SD), cotton stalks (CS) and groundnut shell (GNS) used for all experimental purposes were procured from Raghuvir Timbers, Anand, Gujarat, India; from Sherdi village of Anand, Gujarat, India and from ‘Darshan Seeds Industries’, Modasa, Gujarat, India respectively. The procured materials were grinded and sun-dried for 2 days. The  sun dried materials were screened and then used for further experiments and analyses.

### Methods

#### Batch sorption

For the experiments, different stock solutions containing 25 mg l^−1^, 50 mg l^−1^, 75 mg l^−1^, and 100 mg l^−1^ MB were prepared by dissolving appropriate amount of MB in deionized water. Since biomass has lower sorption capacity as compared to activated carbon and also it is easily available at cheaper rates, 1 g of sorbent was taken for experimental purposes (Gupta and Suhas 2009). MB solution (100 ml) with requisite concentration was taken in a beaker and biomass (1 g) was added into it**.** The prepared solution was isothermally stirred for 180 min at 400 rpm. Afterwards, it was filtered through suction filtration. The filtrate samples were then taken for absorbance measurement using UV–Vis Spectrophotometer (Shimadzu UV-1700 PharmaSpec) at 665 nm wavelength.

Amount of dye removed by sorbents at equilibrium, *q*_*e*_ can be calculated using following equation (Ertaş et al. 2010):

1 $$q_{e} = \frac{{\left( {C_{0} - C_{e} } \right)V}}{W},$$

where *q*_*e*_ is the amount of dye sorbed (mg g^−1^). Furthermore, *C*_*0*_ and *C*_*e*_ are the initial and equilibrium liquid phase concentration of dye (mg l^−1^), respectively. *C*_*e*_ is obtained using absorbance measured from UV–Vis Spectrophotometer during experiments and further using it in the equation obtained from Beer–Lamberts law. *V* (l) is the volume of solution used for experiment and *W* (g) is the weight of sorbent used.

Effects of change in various parameters affecting batch sorption was studied and best sorbent was determined. Furthermore, isotherm study, thermodynamic study, kinetic study and dynamic study of batch sorption was carried out to analyze the biosorption process.

#### Column studies

**Fig. 2:**
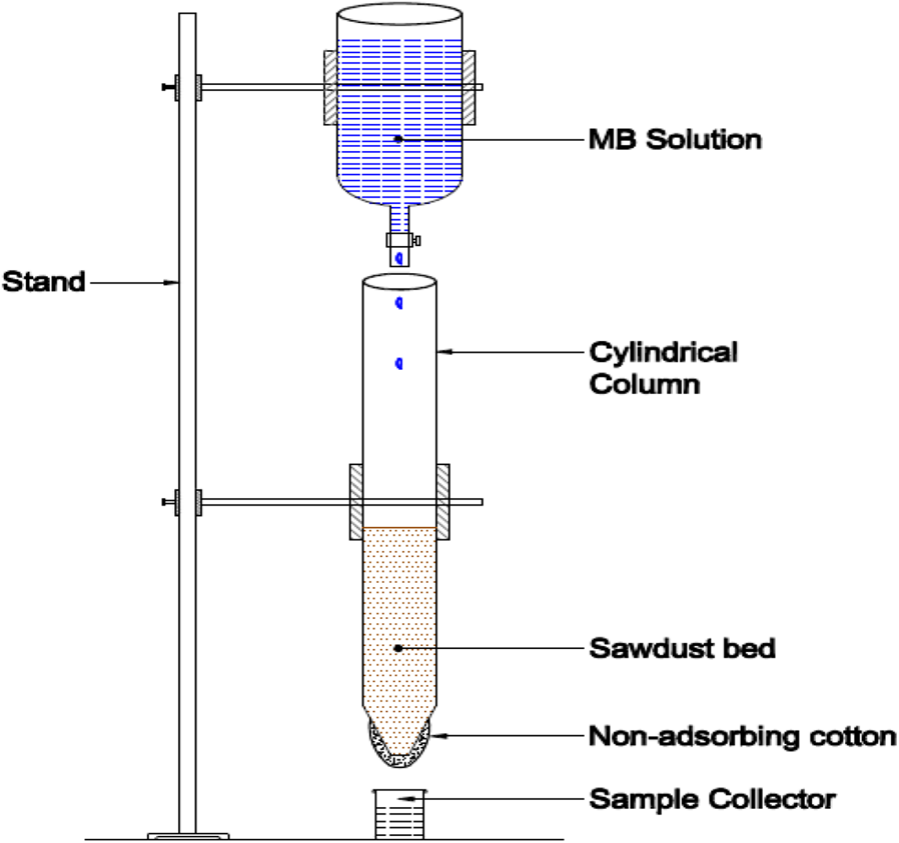
Experimental setup used for column studies

Column studies were performed for SD (0.15–0.3 mm), because it is the best sorbent among the screened biomass resources. Required amount (1 g, 2 g) of SD was loaded into the column for preparing the bed (Fig. 2). The study was carried out at 30 °C in a borosilicate cylindrical column with 1 cm diameter, 1.2 mm thickness and 27 cm height. MB solution (100 ppm) was prepared and filled in the repository arranged above the column. Flowrate (1.5 ml min^−1^, 1.8 ml min^−1^) was measured by adjusting the knob of repository and then it was kept on the top of column; so that, drops fell directly into the bed in-side the column. Non adsorbing cotton was plugged at one end of column to support the SD bed and a sample collector was placed at the bottom of it to collect samples in a fixed time interval. The pictorial representation of the setup is presented in Fig. 2. Column studies were conducted to estimate the breakthrough curve and to ease the scale up procedure. In addition, breakthrough curve modelling was done for predicting breakthrough curve parameters.

## Characterization of sorbents

### Proximate analysis

Proximate analysis (wt%) of the biomass-based sorbents was performed following ASTM standards viz. D-871-82, ASTM E-1755-01 and ASTM E-872 for determination of moisture content, ash content, and volatile matter respectively. In addition, fixed carbon was calculated by difference. These calculations were done on dry basis and the results were obtained for all the sorbents are presented in Table 1.

**Table 1:** Proximate analysis of CS, SD and GNS on dry basis

Material	Proximate analysis (wt%)				Surface area (m^2^ g^−1^)	Total pore volume (cc gm^−1^)	Avg pore diameter (A^0^)
	Ash	Volatile matter	Fixed carbon	Moisture			
CS	5.21	80.73	9.67	4.39	1.8516	0.0029	62.64
GNS	3.09	79.43	13.20	4.28	1.5126	0.0018	47.60
SD	8.34	83.63	6.31	1.72	1.0054	0.0010	39.78

## Results and discussion

### Batch sorption studies

#### Effect of initial concentration on dye removal in batch sorption studies

Effect of initial dye concentration on the dye removal for 1 g (0.15–0.3 mm) sorbent at 30 °C, 45 °C and 60 °C is studied here. With increase of the initial dye concentration (*C*_*0*_) driving force acting on dye molecules moving towards sorbent increases and it can overcome resistance offered by the film easily. In addition, increase in initial dye concentration will lead to better occupancy of active sites which might be empty at low dye concentrations.

**Fig. 3:**
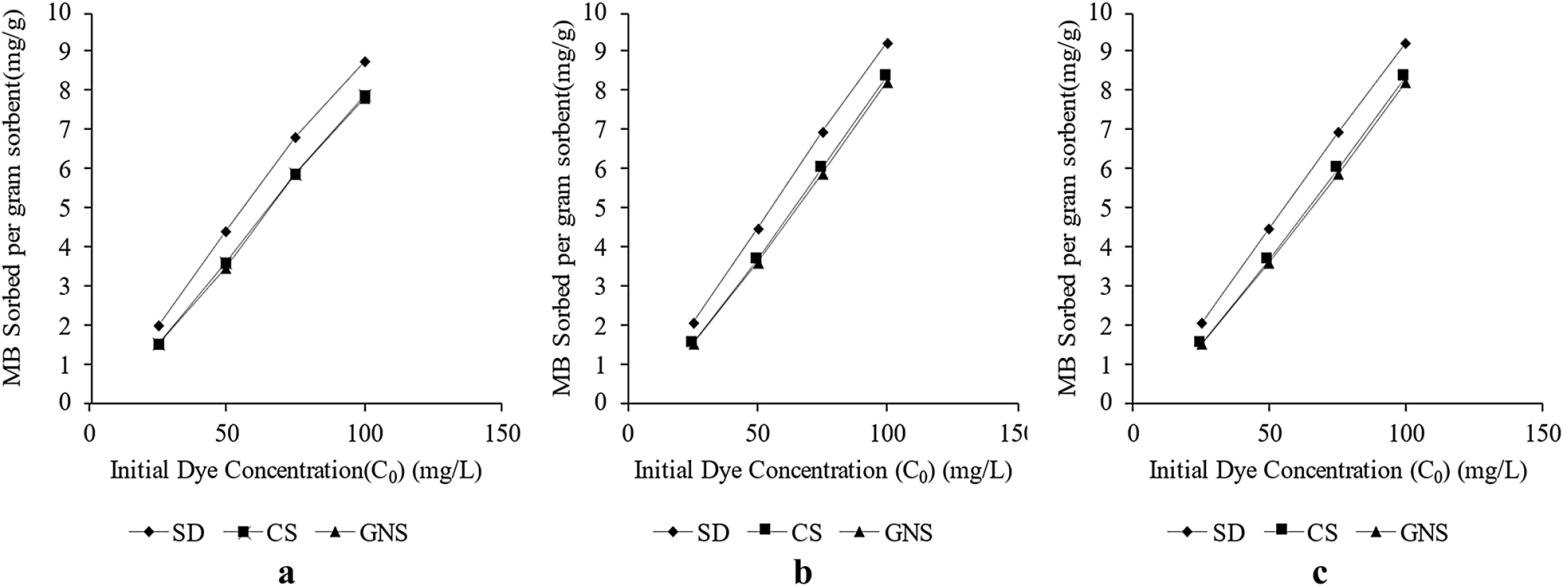
**a** Impact of initial dye concentration on dye removal at 30 °C for SD, CS and GNS; **b** Impact of initial dye concentration on dye removal at 45 °C for SD, CS and GNS; **c** Impact of initial dye concentration on MB removal at 60 °C for SD, CS and GNS

From the experiments it was revealed that equilibrium sorption capacity of SD, CS and GNS changed from 1.88 mg g^−1^ to 8.7 mg g^−1^, 1.47 mg g^−1^ to 7.98 mg g^−1^ and 0.897 mg g^−1^ to 7.96 mg g^−1^ as initial concentrations were changed from 25 to 100 ppm at 30 °C (Fig. 3a). Similar trends were observed for impact of initial dye concentration on MB sorption capacity at 45 °C (Fig. 3b) and 60 °C (Fig. 3c). The data for all set of variations in initial dye concentration at different temperatures has been quantified and visualized in Fig. 3a–c. Similar trend has been observed by other authors for sorption of methylene blue on activated carbon (Hameed et al. 2007), fly-ash (Basava Rao and Ram Mohan Rao 2006) and cotton stalks (Ertaş et al. 2010).

#### Effect of temperature on dye removal in batch sorption studies

Effect of temperature on dye removal was studied using 1 g of SD, CS and GNS, whose particle size falls in 0.15–0.3 mm, as sorbents. Studies were carried out at 30 °C, 45 °C and 60 °C. The sorption capacity increased with increase in temperature. Therefore, dye sorption was an endothermic process. Increasing temperature enhances the rate of diffusion of sorbate molecules across the boundary layer and further towards the pores of sorbent material. Moreover, decrease in viscosity with the temperature in liquids can make the diffusion movement fast by reducing the shear observed by molecules of adsorbate during mass transfer. In addition, it can be said that altering the temperature will lead to change in equilibrium concentration (*C*_*e*_) of adsorbent for specific adsorbate.

**Fig. 4:**
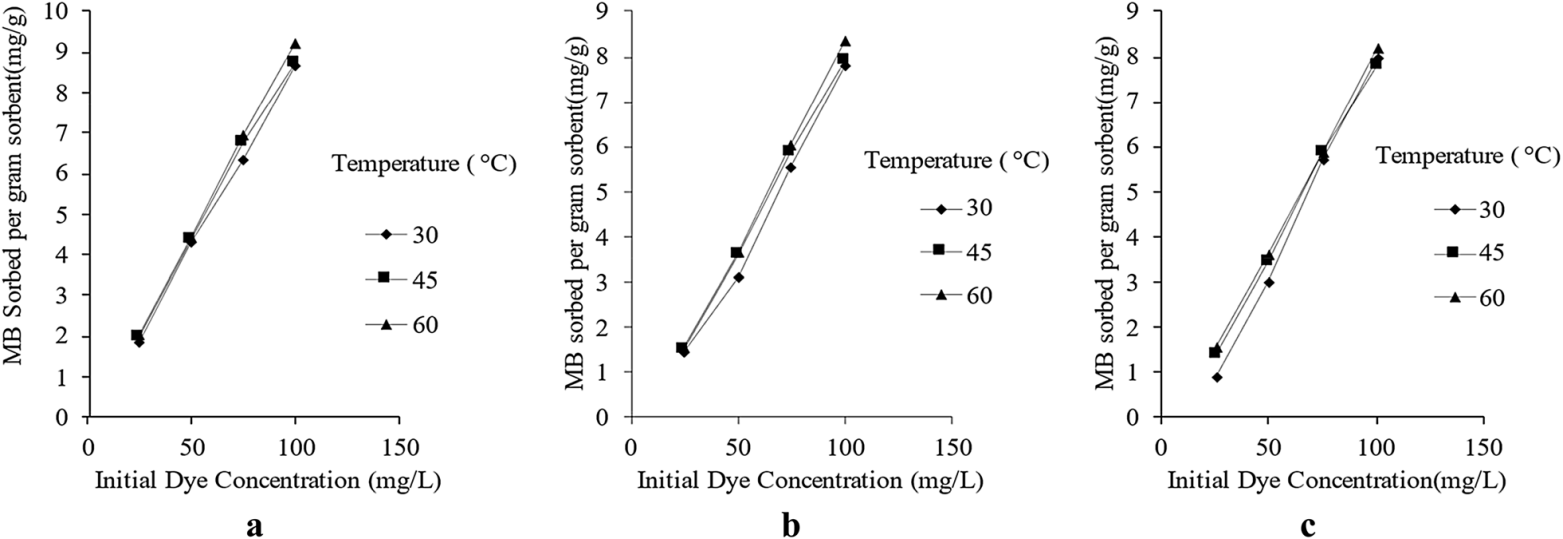
**a** Impact of temperature on the MB removal for SD at 30 °C, 45 °C and 60 °C; **b** Impact of temperature on the MB removal for CS at 30 °C, 45 °C and 60 °C; **c** Impact of temperature on the MB removal for GNS at 30 °C, 45 °C and 60 °C

It was noted that sorption capacity of SD, CS and GNS changed from 8.72 to 9.22 mg g^−1^, 7.89 mg g^−1^ to 8.37 mg g^−1^ and 7.8 mg g^−1^ to 8.2 mg g^−1,^ respectively, as the temperature was raised from 30 to 60 °C for 100 ppm MB solution. Similar trend was obtained for all set of initial dye concentration for all sorbents as temperature was changed from 30 to 60 °C. Results obtained for different sorbents were quantified and presented in Fig. 4a–c. Other authors also observed similar trend for bamboo charcoal (Zhu et al. 2009), sepiolite (Doǧan et al. 2007) and cotton waste (Doǧan et al. 2007; Ertaş et al. 2010).

#### Effect of particle size on dye removal in batch sorption studies

Intra-particle diffusion rate constant (*K*_*d*_) and intra-particle diffusivity (*D*) are related in the following way:

2 $$K_{d} = \frac{{6 q_{e} }}{R}\sqrt {\frac{D}{\pi }} ,$$

where *R* (cm) is the particle radius and *q*_*e*_ (mg g^−1^) is the solid phase concentration at equilibrium. The equation clearly states that *K*_*d*_ is inversely dependent on adsorbent particle’s radius (*R*). The increase in dye removal with decreasing particle size/radius is expected as small particles (0.15–0.3 mm) have larger external surface area; this increases adsorption probability of adsorbent on adsorbate. As a result, it causes higher dye removal for small particle sizes as compared to large particles (0.85–2 mm).

**Fig. 5:**
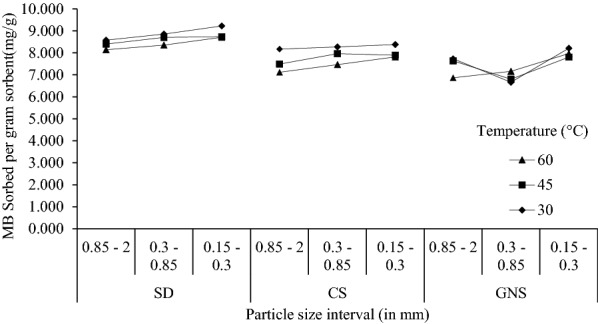
Sorption of MB on various particle sizes of SD, CS and GNS at 30 °C, 45 °C and 60 °C

We have examined the effect with biomass particles obtained after sieving from four different mesh size (ASTM) namely 10, 20, 50 and 100. The particles were divided on the basis of their particle size into three different classes 0.85–2 mm, 0.3–0.85 mm and 0.15–0.3 mm. Experiments were performed for biomass particle collected in above mentioned ranges of particle size. Taking 1 g biomass particle as sorbent with 100 ppm MB solution, experiments were carried out at 30 °C, 45 °C and 60 °C. Keeping the rest of the parameters constant as mentioned in the batch sorption methods, the results we obtained are as follows:

Sorption increases with deceasing in particle size as presented in Fig. 5 for SD particles falling in 0.15–0.3 mm, it is 9.21 mg g^−1^ which is 7.5% more than SD particles falling in 0.85–0.2 mm at similar conditions. For CS particles falling in 0.15–0.3 mm sorption is 8.37 mg g^−1^ at 60 °C; whereas, it is 8.16 mg g^−1^ for CS particles falling in 0.85–0.2 mm under similar experimental conditions (Fig. 9). The dye sorption observed for GNS particles falling in 0.85–0.2 mm at 60 °C is 6.864 mg g^−1^; whereas, 7.96 mg g^−1^ was obtained for GNS particles falling in 0.15–0.3 mm at same conditions (Fig. 9). Moreover, we can see 6–8% rise in sorption capacity as particle size decreases. This supports the fact that surface area of sorbent plays an important role in sorption of dye molecules. In addition, small particles move faster in solution while stirring as compared to larger particles; thus, increasing mass transfer. Moreover, from the studies it has been confirmed that the boundary layer thickness is lesser for small particles which shows lesser resistance to mass transfer (Ong et al. 2007). Other authors reported similar trend while performing experiments on rice hull (Ong et al. 2007) and on bamboo charcoal (Zhu et al. 2009).

#### BET analysis

Brunauer–Emmett–Teller (BET) analysis was carried out to determine the pore size and pore volume of the sorbents used (Table 2). As discussed previously, intra-particle diffusion rate constant (*K*_*d*_) is inversely proportional to the radius of sorbent used. From BET analysis, SD was seen having the lowest pore diameter and surface area. It can be inferred that low pore diameter will accommodate more pores for a given surface area which will add to diffusion rate. In addition, the small size of SD compared to CS and GNS will have lesser boundary layer thickness which will not obstruct the movement of sorbate.

**Table 2:** BET analysis results for SD, CS and GNS

Sorbent	Surface area	Total pore volume	Avg pore diameter
	m^2^ g^−1^	cc gm	A^0^
SD	1.0054	0.001	39.78
CS	1.8516	0.0029	62.64
GNS	1.5126	0.0018	47.6

### Isotherms study

#### Freundlich isotherm

The Freundlich adsorption isotherm explains the reversible and non-ideal adsorption process. Unlike Langmuir isotherm, Freundlich isotherm is not restricted to monolayer adsorption. This empirical relationship describes multilayer adsorption of heterogeneous systems and assumes that specific site has specific adsorption energy involved. The Freundlich isotherm can be expressed as

3 $$q_{e} = kC_{e}^{{\left( \frac{1}{n} \right)}} .$$

Linear equation can be obtained by taking natural logarithm on both sides:

4 $$\ln q_{e} = \ln k + \frac{1}{n}\ln \left( {C_{e} } \right),$$

where *q*_*e*_ is the amount of dye sorbed at equilibrium (mg g^−1^), *C*_*e*_ is the equilibrium concentration of dye in solution (mg l^−1^), *k* and *n* are Freundlich constants which depicts capacity and intensity of sorption, respectively.

**Fig. 6:**
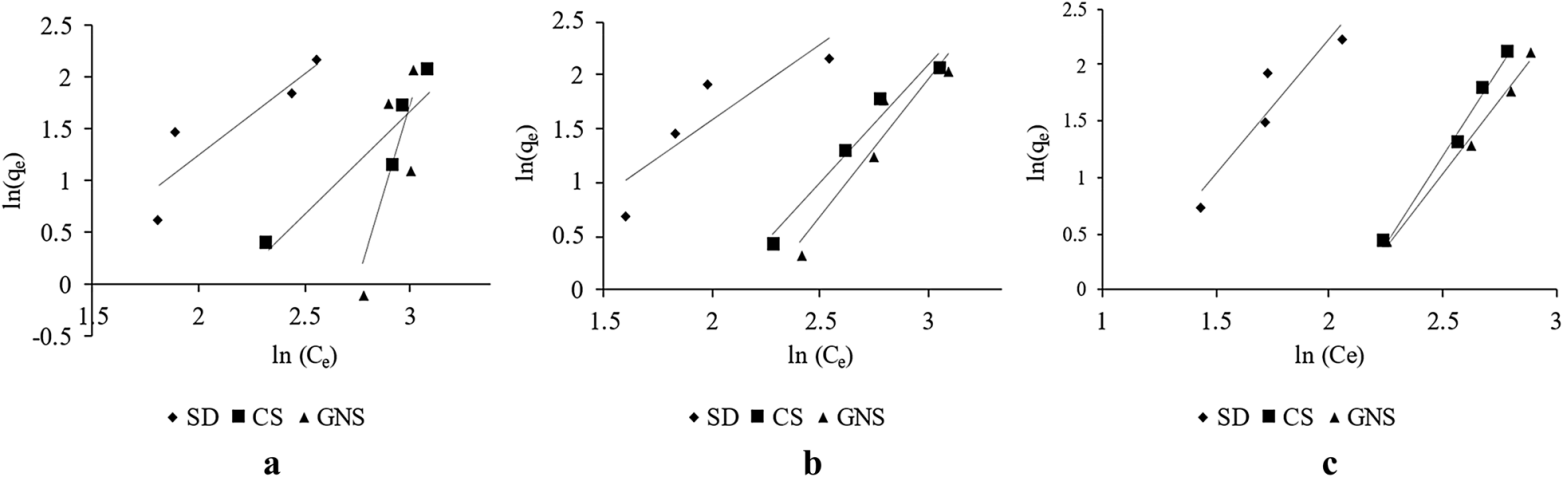
**a** Freundlich isotherm at 30 °C for SD, CS and GNS; **b** Freundlich isotherm at 45 °C for SD, CS and GNS; **c** Freundlich isotherm at 60 °C for SD, CS and GNS

Figure 6a–c shows the Freundlich adsorption isotherm for SD, CS and GNS at three different temperatures, namely, 30 °C, 45 °C and 60 °C for 100 ppm MB solution and 1 g (0.15–0.3 mm) of biomass sorbent. As seen in figures the value of *R*^2^ was noted to check the fitting of Freundlich isotherm. *R*^2^ value ranges from 0.60 to 0.87 for isotherms at 30 °C, 0.76 to 0.96 for isotherms at 45 °C and 0.86 to 0.99 for isotherms at 60 °C (Table 3).

**Table 3:** Values of Freundlich constants *k*, 1/*n* and *R*^2^ for SD, CS and GNS at 30 °C, 45 °C and 60 °C

Temperature (°C)	Biomass	Freundlich Constants		
		1/*n*	*k* (mg g^−1^)	*R*^2^(Freundlich)
30	SD	1.557	0.156	0.797
	CS	1.993	0.013	0.872
	GNS	6.712	0.000	0.602
45	SD	1.398	0.299	0.763
	CS	2.206	0.011	0.964
	GNS	2.589	0.003	0.915
60	SD	2.405	0.075	0.865
	CS	3.102	0.001	0.989
	GNS	2.591	0.004	0.992

The values of parameters *k* and 1/*n* were derived from isotherms plotted and tabulated as below. Freundlich isotherm being one of the simplest models to represent the adsorption phenomena, many times it is not able to determine the constants properly as in the case of GNS at 30 °C. In this case, value of k tends to zero as the value of 1/*n* is very high and it is difficult to visualize such processes using simple isotherms. Hence, to obtain a better fit we used Sips isotherm model which has three parameters and tried to obtain results from it.

#### Sips isotherm

The Sips isotherm was derived from limiting behavior of Langmuir and Freundlich isotherms. This model is valid for localized adsorption without adsorbate–adsorbate interaction (Al-Ghouti and Da’ana 2020). When *C*_*e*_ approaches low value, Sips isotherm reduces to Freundlich, whereas for high values of *C*_*e,*_ it reduces to a monolayer Langmuir isotherm. Operating conditions such as altering of concentration and temperature governs the parameters of Sips isotherm. The sips linear model equation is presented below as

5 $$\frac{1}{{q_{e} }} = \frac{1}{{Q_{\max } \cdot K_{s} }}(\frac{1}{{C_{e} }})^{{\left( \frac{1}{n} \right)}} + \frac{1}{{Q_{\max } }},$$

where *K*_*s*_ (mg^−1^) and *Q*_max_ (mg g^−1^) are the sips equilibrium constant and maximum adsorption capacity values, respectively. *n* whose value ranges from 0 to 1 is dimensionless heterogeneity factor which describe systems heterogeneity. When *n* = 1 Sips isotherm reduces to Langmuir isotherm, and it implies a homogenous adsorption process. Since Sips model can have many solutions, it is difficult to find the exact solution but most probable solution can be obtained by varying n from 0 to 1 and finding the best fit line.

The main motive of using Sips model was to better fit the isotherm process happening here; and it can be seen by improvement in the value of *R*^2^. Here, *R*^2^ (sip) is the value of *R*^2^ obtained from Sips model; whereas, *R*^2^ (Freundlich) is the value of *R*^2^ obtained from Freundlich isotherm model. % Improvement is defined as

6 $$\% {\text{Improvement}} = \frac{{\left( {R^{2} \left( {{\text{sips}}} \right) - R^{2} \left( {{\text{freundlich}}} \right)} \right)}}{{R^{2} \left( {{\text{freundlich}}} \right)}} \times 100.$$

**Fig. 7:**
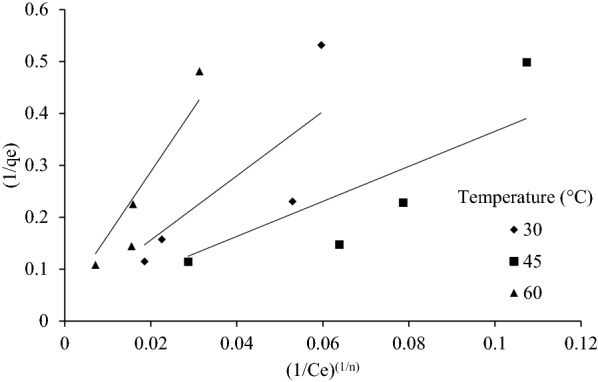
Sips isotherm for SD at 30 °C, 45 °C and 60 °C

**Fig. 8:**
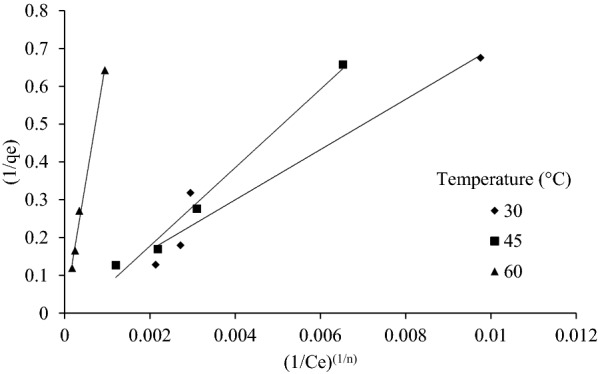
Sips isotherm for CS at 30 °C, 45 °C and 60 °C

As tabulated the %Improvement values is greater than 1 in all the cases except one, which shows that Sips isotherm model is better fit as compared to Freundlich isotherm model (Table 4). Sips isotherm for SD and CS are shown in Figs. 7 and 8, respectively.

**Table 4:** Value of Sips constants for SD, CS and GNS at 30 °C, 45 °C and 60 °C and %Improvement due to Sips isotherm

Temperature (°C)	Biomass	Sips constants				Comparison	
		1/*n*	*Q*_max_ (mg g^−1^)	*K*_*s*_ (mg^−1^)	*R*^2^ (sip)	*R*^2^ (Freundlich)	%Improvement
30	SD	1.557	31.250	0.005136	0.697	0.797	− 12.463
	CS	1.993	29.888	0.000503	0.938	0.871	7.5829
	GNS	6.712	45.454	2.2E−10	0.856	0.602	42.155
45	SD	1.398	35.714	0.00587	0.802	0.763	5.137
	CS	2.206	50.012	0.000192	0.988	0.964	2.521
	GNS	2.589	33.333	9.15E−05	0.973	0.915	6.237
60	SD	2.405	23.809	0.00341	0.932	0.865	7.725
	CS	3.102	78.614	1.89E−05	0.994	0.989	0.414
	GNS	2.591	95.708	4.68E−05	0.997	0.992	0.494

The values of *Q*_max,_ *K*_*s*_, *R*^2^ and 1/*n* obtained from Sips model for all the isotherms were quantified and tabulated below (Table 4).

### Thermodynamics of sorption

**Fig. 9:**
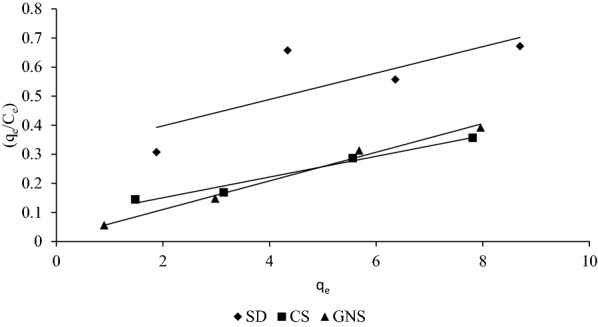
(*q*_*e*_/*C*_*e*_) vs *q*_*e*_ plot to obtain *K*_*c*_ for sorption of MB on SD, CS and GNS

**Fig. 10:**
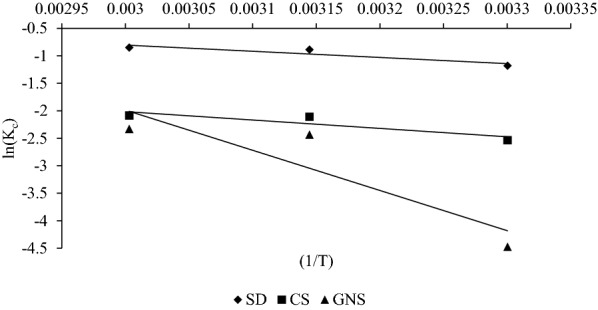
Van’t hoff plots for MB sorption onto SD, CS and GNS

Sorption thermodynamics were determined using thermodynamic equilibrium coefficient obtained at different temperature and concentration to obtain various thermodynamic parameters. The value of Gibs free energy (∆*G*°) for sorption can be calculated as

7 $$\Delta G^{0} = - RT\ln K_{c} ,$$

where *K*_*c*_ is thermodynamic equilibrium constant (l/g) for sorption process. According to (Niwas et al. 2000), *K*_*c*_ can be obtained by plotting (*q*_*e*_/*C*_*e*_) vs q_e_ (Fig. 9) and extrapolating *q*_*e*_ to 0. Van’t Hoff equation which is mentioned below establishes relation between ∆*H*°, ∆*S*° and *K*_*c*_ was used to obtain ∆*H*° and ∆*S*° for the sorption (Yadava et al. 1991; Namasivayam and Kavitha 2002; Hameed and El-Khaiary 2008):

8 $$\ln K_{c} = \frac{{ - \Delta H^{0} }}{{{\text{RT}}}} + \frac{{\Delta S^{0} }}{R}.$$

ln (*K*_*c*_) vs (1/*T*) was plotted in Fig. 10 and slope of the line obtained was used to find ∆*H*° and intercept was used to find ∆*S*°. The thermodynamic parameters obtained from the calculations are shown in Table 5 and *R*^2^ mentioned there is the square of correlation coefficient for ln (*K*_*c*_) vs (1/*T*). It was observed that ∆*H*° > 0 which indicates that the sorption process is endothermic in nature. In addition, the value of *K*_*c*_ increases with increase in temperature which too corroborates that process is endothermic in nature (Table 5). Moreover, the value of ∆*G*° was found to be decreasing with increase in temperature which shows that spontaneity increases with temperature. The positive value of ∆*S*° shows increased randomness at solid/liquid interface during the sorption of dye on SD, CS and GNS. Similar trend in thermodynamic parameters have been described for adsorption of Congo red onto activated carbon prepared from coirpith (Namasivayam and Kavitha 2002).

**Table 5:** Values of thermodynamic parameters and equilibrium constants for sorption of MB onto SD, CS and GNS at 30 °C, 45 °C and 60 °C

Sorbent	Temperature (°C)	*K*_*c*_ (L/g)	∆*G*° (J mol^−1^)	∆*S*° (J mol^−1^)	∆*H*° (J mol^−1^)	R^2^
SD	30	0.307	2974.874	21.378	9356.99	0.855
	45	0.411	2348.241			
	60	0.427	2353.374			
CS	30	0.079	6384.809	21.310	12,690.41	0.809
	45	0.121	5577.175			
	60	0.124	5777.069			
GNS	30	0.011	11,271	165.764	60,763.05	0.808
	45	0.087	6434.695			
	60	0.097	6450.628			

### Kinetics of sorption

A kinetic study provides the idea about reaction time and estimates the parameter affecting reaction equilibrium. Various authors studied kinetics of MB sorption on different sorbents including activated carbon and concluded that pseudo-second-order kinetic model is the most suitable kinetic model for biosorption (Bello et al. 2008; Chowdhury and Saha 2010; Simonin 2016; Guarín et al. 2018). Kinetic studies were performed at 30 °C using 1 g (0.15–0.3 mm) of sorbent and keeping rest of the condition same as discussed in the methods section.

#### Pseudo-second-order Kinetics

**Fig. 11:**
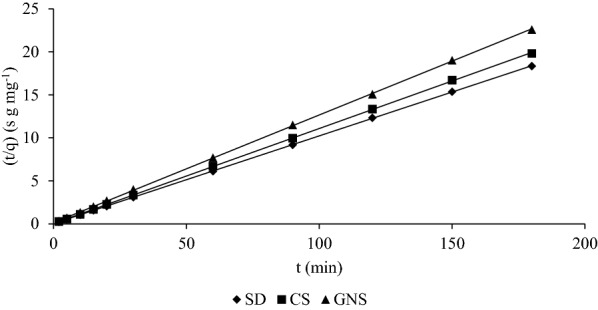
Plot of (*t*/*q*) vs *t* for 1 g SD, CS and GNS at 30 °C

Pseudo-second-order kinetics being the widely applicable kinetic model for biosorption processes is discussed here. (Ho and McKay 1999) proposed linear form of pseudo-second-order kinetic equation which is represented as

9 $$\frac{t}{q} = \frac{t}{{q_{e} }} + \frac{1}{{k_{2} q_{e}^{2} }},$$

where *q* is the sorption rate (mg g^−1^ of MB) on sorbent at any time *t* and *q*_*e*_ is the concentration when the sorption reaches equilibrium. (*t/q*) vs *t* was plotted in Fig. 11 and the value of *q*_*e*_ is obtained which is shown in the Table 6.

**Table 6:** q_e_ and R^2^ values obtained for SD, CS and GNS from pseudo second order model

Sorbent	*q*_*e*_ (mg g^−1^)	*R*^2^
SD	9.8135	0.9999
CS	9.0497	0.9999
GNS	7.9872	0.9999

Values of *q*_*e*_ were obtained from the line plotted in Fig. 11 which are near to the ones obtained previously in batch sorption and also the *R*^2^ value is 0.9999 which shows that this model is best fit for biosorption processes. 9.81 mg g^−1^, 9.04 mg g^−1^ and 7.98 mg g^−1^ are the equilibrium concentration obtained for SD, CS and GNS, respectively, while it was 8.75 mg g^−1^, 7.982 mg g^−1^ and 7.92 mg g^−1,^ respectively, as obtained experimentally for the same.

### Sorption dynamics

When sorbent is added into MB solution the mass transfer occurred can be conveyed explicitly in four stages (Chen et al. 2020b; Li et al. 2018; Zhou et al. 2019; Zhang et al. 2018)—(i) Diffusion of dye molecules from liquid bulk to the liquid film surrounding adsorbent. (ii) Diffusion from film to adsorbent surface. (iii) Diffusion through pores of the adsorbent. (iv) Uptake of adsorbate on the active sites. Based on the resistance offered in different steps kinetic modelling of diffusion can be done. Steps (i) and (ii) are combined known as film diffusion, whereas (iii) and (iv) are comes under particle diffusion.

**Fig. 12:**
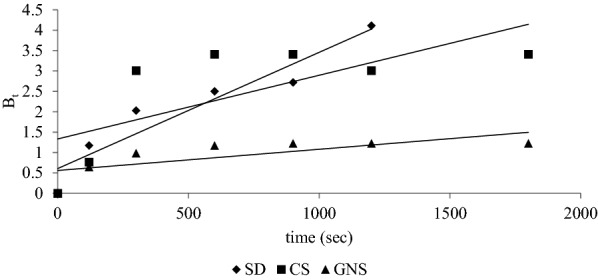
B_t_ vs t at 30 °C for 1 g of SD, CS and GNS

To determine the governing diffusion mathematically Boyd et al. (Mittal et al. 2009) suggested a mathematical treatment, where fractional attainment *F *of equilibrium at any time t was required and the relations given below were proposed:

10 $$F = 1 - \frac{6}{{\pi^{2} }}\mathop \sum \limits_{1}^{\infty } \frac{1}{{n^{2} }}\exp \left( { - n^{2} \cdot B_{t} } \right)$$

11 $$F = \frac{{q_{t} }}{{q_{e} }},$$

where *q*_*t*_ and *q*_*e*_ are the amount sorbed after time *t* and amount sorbed at equilibrium, respectively:

12 $$B_{t} = \frac{{\pi^{2} D_{i} }}{{r_{0}^{2} }} = {\text{time constant,}}$$

where *D*_*i*_ is diffusion coefficient, *B*_*t*_ is time constant and *r*_0_ is the radius of spherical sorbent particle. *B*_*t*_ values were obtained from *F* values using table proposed by Reichenberg (1953). Rate controlling diffusion step can be determined using B_t_ vs t graphs. *B*_*t*_ vs *t* graph was plotted for SD, CS and GNS, as shown in Fig. 12. The *B*_*t*_ vs *t* graph shows that the nature of change can be better represented by line which does not passes through origin for SD, CS and GNS. As studied by Reichenberg, it can be inferred from the nature of curve that governing diffusion process is film diffusion which depicts that maximum resistance is observed by sorbate while reaching the surface of sorbent. Mittal et al. (2009) obtained similar results for sorption of anionic dye congo-red using waste material.

### Breakthrough curves and modelling of column studies

From the studies we obtained that SD has the highest sorption capacity for MB compared to CS and GNS. It is well known that column studies are more effective compared to batch systems as exhaustion capacity of column is usually relatively higher. Moreover, fixed bed column studies are easy to perform and can be effectively scaled up for further usages. Breakthrough curves were plotted to check the applicability of SD as sorbent on higher scale. Column studies give clear idea about the behavior of sorbent when exposed to a continuous system. Hence column studies were performed with SD as sorbent to describe fixed bed column behavior and to scale up it for industrial purposes. Three models namely Thomas, Adam–Bohart and Yoon–Nelson were used to obtain kinetic model of column studies and predict breakthrough curves.

#### Breakthrough curves

**Fig. 13:**
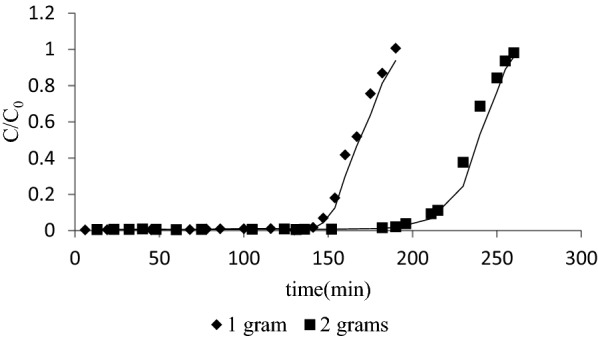
Breakthrough curves obtained from column study of 1 g and 2 g SD beds

Breakthrough curves are the plots which help to analyze the behavior of bed with respect to time. Two beds containing 1 g SD and 2 g SD were taken for column studies and the flow rates were kept 1.5 ml min^−1^ and 1.8 ml min^−1,^ respectively. *C* is the effluent concentration at any time *t* and *C*_0_ is the influent concentration. Here we have plotted (*C*/*C*_0_) vs time and the results obtained are shown in Fig. 13 for 1 g SD and 2 g SD. From Fig. 13, it can be seen that there is a sharp change in *C*/*C*_0_ after 145 min and 200 min for bed with 1 g SD and 2 g SD, respectively, which shows that the bed is on the threshold of saturation. The point of sharp change is called breakthrough point, the effluent concentration at breakthrough point is called breakthrough concentration (*C*_*b*_) and the time at which it happens is called breakthrough time (*T*_*b*_). C vs t was plotted for columns with 1 g SD and 2 g SD and area of curve was measured to obtain the amount of MB sorbed by the column. 24.02 mg and 34.5 mg MB was sorbed by columns with 1 g SD and 2 g SD, respectively. Breakthrough time for 1 g SD and 2 g SD bed obtained at *C*/*C*_0_ = 0.1 was found to be 145 min and 200 min, respectively. 200 ml and 350 ml MB solution was treated by bed till breakthrough by 1 g SD bed and 2 g SD bed, respectively.

#### Thomas model

Thomas Model is one of the most general and widely used models for describing the behavior of sorbents in biosorption process. It assumes plug flow behavior in bed and the equation employed is expressed as

13 $$\ln \left( { \frac{{C_{0} }}{{C_{t} }} - 1} \right) = \frac{{K_{Th} q_{0} m}}{Q} - K_{Th} C_{0} t,$$

where *K*_th_ is Thomas model constant (ml min^−1^ g^−1^), *q*_*o*_ (mg g^−1^) is the sorption capacity of sorbent, *m* is the mass of sorbent in the column and *Q* (ml min^−1^) is the flow rate of MB solution being poured from the top into the column. $\mathrm{Ln}\left( \frac{{C}_{0}}{{C}_{t}}-1\right)$ vs *t* was plotted and the line equation was used to obtain *K*_th_ and *q*_0._ The values of *q*_0_ increases with decrease in flow rate and bed height. The value of q_0_ was observed to be 28.93 mg g^−1^ and 19.44 mg g^−1^ for 1 g SD column and 2 g SD column, respectively (Table 7). It can be seen that column studies are effective compared to batch sorption methods as sorption capacity observed for SD is more in column studies setup (19–29 mg g^−1^) compared to batch sorption setup (7–10 mg g^−1^). (Lakshmipathy and Sarada 2016) obtained the similar trend for Thomas model.

**Table 7:** Values of parameters obtained from Thomas model, Yoon–Nelson model and Adam–Bohart model

Column studies parameters		Thomas		Yoon–Nelson		Adam–Bohart	
Bed height (cm)	Flow rate (ml min^−1^)	*K*_Th_ (ml min^−1^ mg^−1^)	*q*_0_ (mg g^−1^)	*K*_YN_ (min^−1^)	$\tau$ (min)	*k*_AB_ (L mg^−1^ min^−1^)	*N*_0_ (mg l^−1^)
5.7	1.5	0.00038	28.93	0.0376	195.09	0.000324	7249.892
10.5	1.8	0.00027	19.44	0.0276	252.09	0.000199	6909.366

#### Yoon–Nelson model

Yoon–Nelson developed a simplistic model which assumes that the rate of decrease in the probability of adsorption for each adsorbate molecule is proportional to the probability of sorbate sorption and the probability of sorbate breakthrough on sorbent (Lakshmipathy and Sarada 2016). The linear form of the equation employed in model is as follows:

14 $$\ln \left( {\frac{{C_{t} }}{{C_{0} - C_{t} }}} \right) = K_{{{\text{YN}}}} - \tau K_{{{\text{YN}}}} ,$$

where *K*_YN_ (min^−1^) is Yoon–Nelson proportionality constant and $\tau$ is time required for retaining 50% of initial sorbate. The values of *K*_YN_ and $\tau$ can be obtained by plotting $\mathrm{ln}\left(\frac{{C}_{t}}{{C}_{0}-{C}_{t}}\right)$ vs *t*. The values of *K*_YN_ and $\tau$ obtained for all breakthrough curves are tabulated in Table 7. The values of $\tau$ were found to increase with increase in flowrate and bed height. Here the value of $\tau$ was found to be 195 min for bed with height 5.7 cm and 252 min for bed with height 10.5 cm. The values of $\tau$ obtained from breakthrough curves (192 min and 238 min) and Yoon–Nelson model (195 min and 252 min) are comparable. (Lakshmipathy and Sarada 2016; Chatterjee et al. 2018) obtained similar trend in results of Yoon–Nelson model.

#### Adams–Bohart model

Adams–Bohart model is widely used for prediction of breakthrough curves obtained from column studies. According to this model equilibrium is not instantaneous and rate of sorption is proportional to the fraction of sorption capacity. It is mainly useful in predicting the initial part of breakthrough curve. The linearized equation employed in the model is

15 $$\ln \frac{{C_{t} }}{{C_{0} }} = k_{{{\text{AB}}}} C_{0} t - k_{{{\text{AB}}}} N_{0} \frac{z}{{\mu_{0} }},$$

where *C*_0_ is the influent MB concentration, *C*_*t*_ is the effluent MB concentration at any time *t*, *k*_AB_ is the kinetic constant (L mg^−1^ min^−1^), *N*_0_ is the saturation concentration (mg l^−1^), *z* is the bed height (cm) and ${\mu }_{0}$ is superficial velocity (cm min^−1^). $ln\frac{{C}_{t}}{{C}_{0}}$ vs *t* was plotted to obtain the model parameters. The calculated parameters *N*_0_ and *k*_AB_ are tabulated in Table 7. It was observed that the value of *N*_0_ decreases with increase in bed height and flow rate. Here the value of *N*_0_ was obtained to be 7249.89 mg l^−1^ in case of bed height 5.7 cm and 6909.366 mg l^−1^ in case of bed height 10.5 cm.

### Scale-up procedure

A column that can run with SD as a sorbent was considered to remove MB (or similar) dyes from the waste water/aqueous solution assuming that the column behaves similarly to the column used in the experiments. The widely used column size for water treatment using activated carbon as a sorbent in medium scale industry has diameter in the range 50 cm–100 cm and generally selected by customers as per their requirement.

**Fig. 14:**
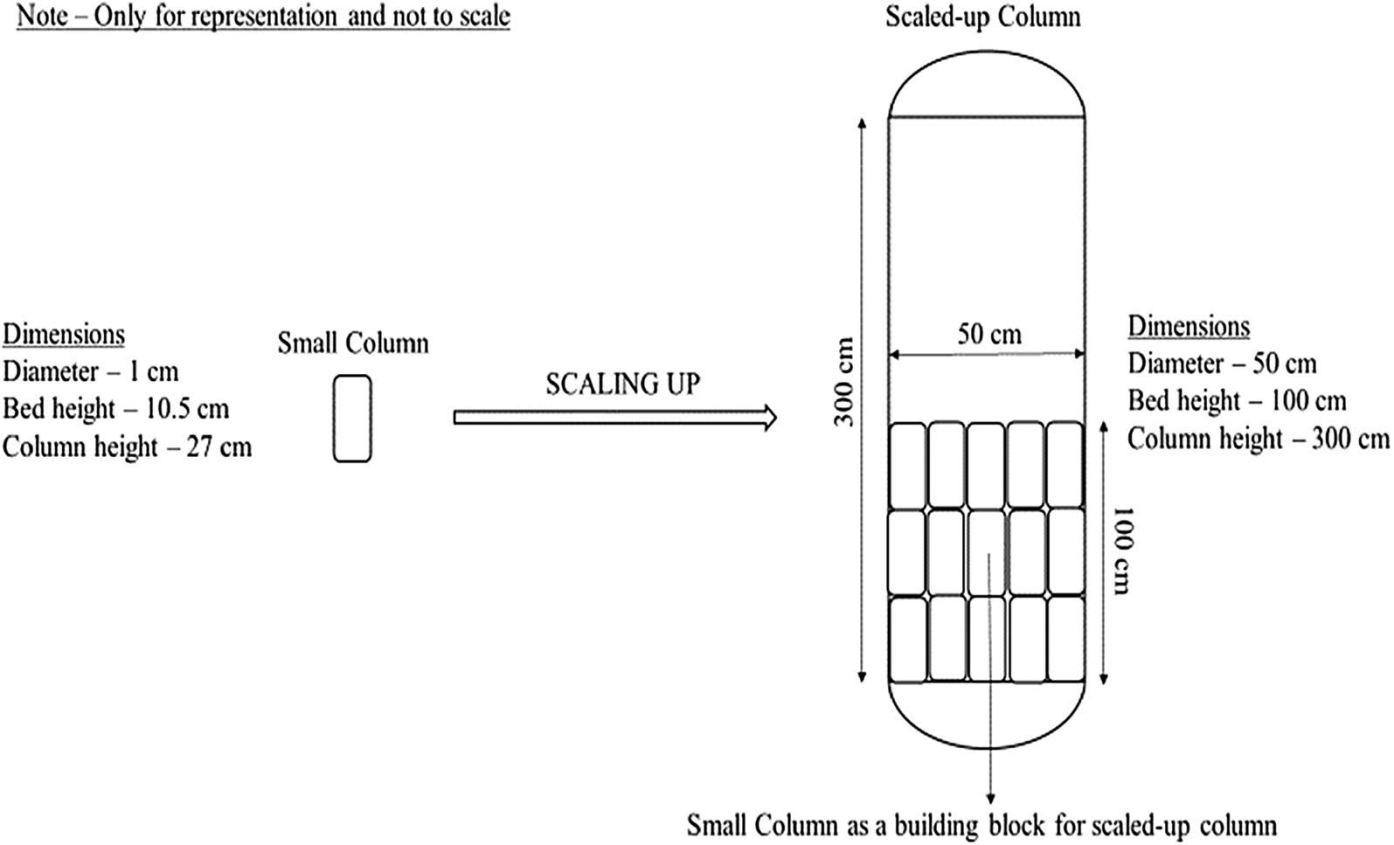
Proposed scaled-up column design along with dimensions

An up-scaled cylindrical sorption column with 50 cm diameter and 300 cm height containing SD bed of height 100 cm was designed. Clearance of 200 cm (300–100 = 200 cm) above bed is necessary as wastewater may stagnate on top of the bed while starting the column. The idea for scaling up is shown in the figure, where small columns are used as a building block for up-scaling (Fig. 14).

Bed height of small column (*H*_1_) = 10.5 cm

Diameter of small column (*D*_1_) = 1 cm

Height of small column (*L*_1_) = 27 cm

Weight of SD used in small column (*W*_1_) = 2 g

Volume of small column (*V*_1_) = 8.24 cm^3^

Cross sectional area of small column (*C*_1_) = 0.78 cm^2^

Flow rate in small column (*F*_1_) = 1.8 ml min^−1^

Breakthrough time for small column (*T*_b1_) = 200 min = 3.33 h

Maximum volume of water treatment possible in small column (CA_1_) = 350 ml

Bed height of scaled up column (*H*_2_) = 100 cm

Diameter of scaled up column (*D*_2_) = 50 cm

Height of scaled up column (*L*_2_) = 300 cm

Volume of scaled up column (*V*_2_) = 196,349 cm^3^

Cross sectional area of scaled up column (*C*_2_) = 1963.49 cm^2^:

$$\text{Volume factor }\,\left({V}_{f}\right)=\frac{{V}_{2}}{{V}_{1}}=23,829$$

16 $${\text{Therefore}},V_{1} { } = 23829{ }V_{2} .$$

Thus, a cylindrical column with 50 cm diameter and 100 cm height is similar to 23,829 nos of column with 2 g SD with 10 cm bed height as both of them provide same surface area.

Flow rate of small column = 1.8 ml min^−1:^

17 $${\text{Area}}\;{\text{factor }}\left( {A_{f} } \right) = \frac{{C_{2} }}{{C_{1} }} = 2500.$$

Let flowrate in scaled up column be *F*_2_. Then,

18 $$F_{2} = A_{f} \times F_{1} = 4500\frac{{{\text{ml}}}}{{{\text{min}}}}$$

Hence, the scaled-up column is capable of treating 4500 ml min^−1^ of wastewater.

Let maximum volume of water treatment possible in scaled up column be (CA_2_). Then

19 $${\text{CA}}_{2} = V_{f} \times {\text{CA}}_{1} = 8340150{\text{ ml}}{.}$$

$\mathrm{C}$ Breakthrough time of scaled up column can be obtained in two ways as follows:

20 $$T_{b2 - A} = \frac{{{\text{CA}}_{2} }}{{F_{2} }} = 30.89{\text{ h}}$$

21 $$T_{b2 - B} = T_{b1} \times \frac{{H_{2} }}{{H_{1} }} = 31.71{\text{ h}}{.}$$

Smaller of the ${T}_{b2-A}$ and ${T}_{b2-B}$ was considered for calculation.

Let the amount of SD required for scaled up column be *W*_2_.

Then

22 $$W_{2} = V_{f} \times W_{1} = 47{,}658{\text{ g}}{.}$$

Rate of SD = 3–5 INR per kg (0.04–0.07 USD per kg as on November 6, 2020).

Therefore, ~ 150–250 INR would be the cost of SD required in one cycle of operation.

Assuming 80% efficiency the scale-up column can run for 1 day (30.89 × 0.8 = 24.71 h) and treat 6672 L (8340.15 × 0.8 = 6672 L) wastewater containing dyes which can be used in textile industries. In addition, columns can be designed accordingly for lesser flowrates or arranged in parallel to treat more wastewater. Column regeneration is required after 24 h for maintaining 80% efficiency and the waste SD obtained from used bed can be regenerated using weak (mild) acid as MB is a basic dye. However, regeneration is not advisable for bio-sorbents, because the activity of sorbents reduces after one run and also it is not cost effective, since operational cost for regeneration column is more than purchase cost of SD in bulk. Different strategies involving catalytic degradation can be employed for the removal of MB as an alternative to land filling.

## Conclusion

From the performed experiments it can be said that SD, CS and GNS can be successfully used for removal of MB (basic dye) from aqueous solution. Optimization of experimental conditions shows that dye removal increases with increase in temperature and initial dye concentration, whereas decreases with increase in particle size of adsorbent. The maximum dye removal for SD, CS and GNS follows the trend SD > CS > GNS amongst all the variation studied in experiments. SD, CS and GNS follow Freundlich isotherm; but didn’t follow Langmuir isotherm, which is applicable only for mono-layer adsorption. This indicates that MB sorption onto biomass is a multi-layer adsorption phenomenon. The values of ∆*H*° and ∆S*°* were found to be positive for all sorbents. In addition, the increase in *K*_*c*_ with temperature shows that sorption is endothermic in nature. Decrease in ∆*G*° with temperature depicts that spontaneity decreases with decrement in temperature. Kinetic studies of sorption shows that sorption follows pseudo-second-order kinetics. Adsorption dynamics study concluded that film diffusion is the governing diffusion. Column studies done with SD depicted that it can work better in column rather than batch. Breakthrough curve studies have revealed the potential of SD as a sorbent and it can be used at an industrial scale. Application based scaled-up column design  is proposed with SD as a sorbent which can treat 6.67 tonnes of water in 24 h assuming 80% efficiency. As a result, it can be suggested to use low cost biomass SD over CS and GNS for dye removal from effluents released by textile industries. Preparing a bed of SD and installing it at the output of existing treatment unit can also be better alternative of activated carbon filter (ACF). Not only this, it may be applied in loose form to control small oil/dye spills and to manage the leftover traces of larger oil spills.

## Data Availability

The data that supports the findings of this study are available from the corresponding author upon reasonable request.

## Abbreviations

CPIs: Chemical process industries
SD: Sawdust
CS: Cotton stalks
GNS: Groundnut shell
MB: Methylene blue
BET: Brunauer–Emmett–Teller
ACF: Activated carbon filter
